# Outcomes from Referral to Transplant for Patients with MASLD: A California Liver Network Study

**DOI:** 10.3390/jcm14217841

**Published:** 2025-11-04

**Authors:** Tiffany Y. Lim, Justin A. Steggerda, Hirsh Trivedi, Michael Luu, Aarshi Vipani, Michie A. Adjei, Jasleen Singh, Kali Zhou, Allison Kwong, Monica Tincopa, Irine Vodkin, Veeral Ajmera, Neil Mehta, Chris E. Freise, Mignote Yilma, Ryutaro Hirose, Alexander Kuo, Steven A. Wisel

**Affiliations:** 1 Department of Surgery, Cedars-Sinai Medical Center, Los Angeles, CA 90048, USA; 2 Comprehensive Transplant Center, Cedars-Sinai Medical Center, Los Angeles, CA 90048, USA; 3 Biostatistics Shared Resources, Cedars-Sinai Medical Center, Los Angeles, CA 90048, USA; 4 Department of Medicine, University of California Los Angeles, Los Angeles, CA 90048, USA; 5 Department of Medicine, University of Southern California, Los Angeles, CA 90048, USA; 6 Department of Medicine, Stanford University, Palo Alto, CA 90048, USA; 7 Department of Medicine, University of California San Diego, San Diego, CA 90048, USA; 8 Department of Medicine, University of California San Francisco, San Francisco, CA 90048, USA; 9 Department of Medicine, University of Washington, Seattle, WA 98195, USA

**Keywords:** metabolic dysfunction-associated steatotic liver disease, liver transplantation, transplantation evaluation

## Abstract

**Background:** Metabolic dysfunction-Associated Steatotic Liver Disease (MASLD) is becoming a leading indication for liver transplantation in the United States. In this growing recipient population, the combined effects of underlying liver disease etiology and associated comorbidities on the evaluation pathway to transplantation warrant closer examination of patient outcomes. **Methods:** We analyzed adult liver transplant referrals (*n* = 9981) from the California Liver Network, a multi-center retrospective cohort spanning six high-volume California transplant centers between 2018 and 2020. A total of 6709 patients who underwent formal evaluation were included. Patients were stratified by MASLD vs. non-MASLD etiology and compared for demographics, comorbidities, transplant evaluation timelines, listing rates, and outcomes. **Results:** MASLD patients (*n* = 1477) were older, had higher BMI, and had greater prevalence of metabolic comorbidities than non-MASLD patients (*n* = 5232; *p* < 0.001 for all). Compared to non-MASLD candidates, MASLD patients were more likely to be waitlisted (OR 1.52, 95% CI 1.33–1.74; *p* < 0.001). However, MASLD and non-MASLD patients had no statistically significant difference in the rate of transplant (*p* = 0.182), with clinically similar but statistically inferior post-transplant survival outcomes at 5 years post-transplant (88% vs. 83%; *p* = 0.014). Competing-risk analysis showed that MASLD candidates had higher cumulative incidence of death on the waitlist (*p* < 0.001), although MASLD was not independently associated with waitlist mortality when adjusting for covariates (*p* = 0.300). MASLD patients demonstrated increased mortality following waitlist removal (HR 1.64, 95% CI 1.14–2.35; *p* = 0.008), primarily among those removed for clinical deterioration (HR 1.50, 95% CI 1.01–2.23; *p* = 0.044). **Conclusions:** MASLD patients face unique challenges in liver transplant evaluation. MASLD patients are associated with higher comorbidities, increased incidence of waitlist mortality, and significantly higher mortality rate following waitlist removal. However, transplantation provides significant survival benefit with comparable outcomes to non-MASLD recipients; thus, early access to transplant may optimize outcomes for MASLD liver transplant candidates.

## 1. Introduction

As the prevalence of metabolic syndrome and obesity in the United States continues to rise, the downstream effects on disease-specific presentation continue to proliferate—especially in liver transplantation [1]. Since 2018, Metabolic dysfunction-Associated Steatotic Liver Disease (MASLD) has quickly emerged as the second most common etiology of liver disease, trailing only alcohol-associated liver disease [2]. Similarly, MASLD is the second most common indication for liver transplant in the United States [3], and has been the most rapidly increasing indication for liver transplantation, in both patients with and without hepatocellular carcinoma [4]. The impact of MASLD on liver transplant is only poised to increase, with MASLD-related waitlist addition predicted to increase by 55.4% between 2016 and 2030 [5].

Metabolic syndrome increases the complexity of patient management across the transplant spectrum, as optimal patient management must simultaneously address liver disease and the comorbidities of metabolic syndrome—including diabetes mellitus, hypertension, coronary artery disease, and hyperlipidemia—both pre- and post-transplant [6]. MASLD has been shown to be an independent risk factor for the development of cardiovascular disease, which is a leading cause of mortality in liver transplant patients [7]. While prior national database studies have demonstrated comparable graft and patient survival between MASLD and non-MASLD liver transplant recipients [8], studies have identified metabolic comorbidities as barriers to patients receiving liver transplantation [9]. Therefore, despite comparable transplant outcomes, further studies are needed to distinguish the overall waitlist and transplant trajectories of MASLD and non-MASLD etiologies.

Importantly, the effects of metabolic syndrome, obesity, and associated complications on liver transplant referral and evaluation practice patterns are not well understood. In addition, most current studies utilize large databases and lack the granularity to understand how the particular facets of metabolic syndrome impact a candidate’s likelihood to undergo liver transplant evaluation and subsequently proceed to waitlisting. Here, we utilize the California Liver Network—a research collaborative evaluating all liver transplant referrals over a two-year period at six large volume transplant centers in California—to evaluate the impact of MASLD on patient waitlisting and waitlisting outcomes as compared to non-MASLD candidates. This detailed dataset comprising over 75% of all liver transplant referrals in California enables a granular analysis of patient progression and risk factors, providing potential insights to optimize patient pre-transplant management for liver transplant candidates with MASLD.

## 2. Materials and Methods

**Figure 1:**
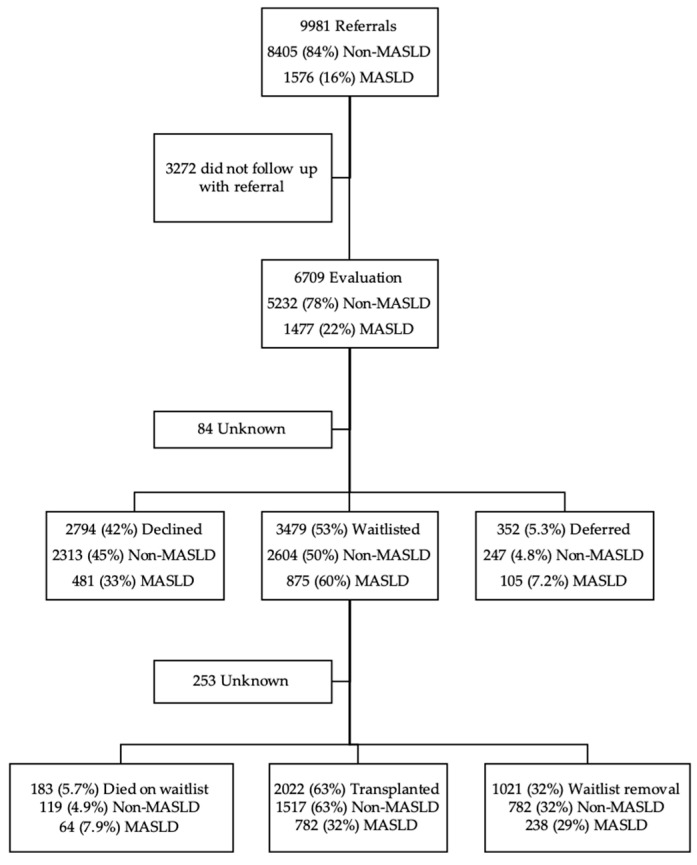
Cohort Selection and Evaluation Outcomes of Patients Referred for Liver Transplantation.

This study utilizes the California Liver Network (CLN), a retrospective consortium of six high-volume liver transplant centers in California (University of California San Francisco, University of California Los Angeles, University of California San Diego, Cedars-Sinai Medical Center, Stanford University, and University of Southern California) formed to investigate referral and evaluation practices in liver transplantation. All adult liver transplant referrals received between 201 January 8 and 31 December 2020 were included in this study (*n* = 9981), with follow-up for outcomes through July 2022. Basic demographic data, including site, age, gender, insurance and address, was obtained for patients who did not initiate evaluation (*n* = 3272). Clinical, demographic, and psychosocial data were retrospectively collected for evaluated patients. Outcomes of evaluation, selection, waitlisting, and transplantation were monitored. Patients were stratified by disease etiology to compare outcomes between MASLD and non-MASLD candidates, as determined by clinical evaluation. Number of patients at each timepoint is shown in Figure 1.

All patients proceeding with liver transplant evaluation within the CLN were included in this study (*n* = 6709). Patients were analyzed for demographics including age, sex, body mass index (BMI), race, MELD, and marital status. Etiology of liver disease was classified as MASLD versus non-MASLD (including alcohol, hepatitis C, hepatitis B, autoimmune hepatitis, primary biliary cirrhosis, primary sclerosing cholangitis, cryptogenic, and other). Candidates were identified as having compensated versus decompensated cirrhosis. Decompensated cirrhosis was defined as events including presence of ascites, hepatic encephalopathy, variceal bleeding, hepatopulmonary syndrome, and portopulmonary syndrome. Comorbidities of metabolic syndrome were likewise captured including diabetes mellitus, hypertension, hyperlipidemia and coronary artery disease requiring intervention.

Baseline patient characteristics were compared between the MASLD and non-MASLD groups using the Wilcoxon rank-sum test for continuous variables and Pearson’s chi-squared test for categorical variables, as appropriate. Missing data for all multivariable analyses were handled using multiple imputation by chained equations (MICE) with five imputations [10]. The imputation model included all variables listed in Table 1. Multivariable analyses were performed on each imputed dataset, and results were pooled using Rubin’s rules [11].

Time-to-event analyses were conducted using both the Cox proportional hazards model and the Fine and Gray subdistribution hazard model. For the outcome of waitlisting, time from waitlist entry to removal, transplantation, or death was analyzed, treating these as competing events. Cumulative incidence functions were estimated for each outcome, and cumulative incidence curves between MASLD and non-MASLD groups were compared using Gray’s test [12]. The Fine and Gray model was used to assess factors associated with each competing outcome, adjusting for relevant patient and clinical covariates.

Factors associated with waitlisting were investigated using a generalized linear mixed-effects model with a random intercept for transplant center. Covariates included age, sex, transplant site, BMI, MELD at listing, presence of comorbidities, and etiology of liver disease.. To assess potential effect modification by BMI class, site, and sex on the association between MASLD and waitlisting, interaction terms were included in the model, adjusting for the same covariates.

Overall survival following waitlist removal was estimated using the Kaplan–Meier method and compared between MASLD and non-MASLD groups using the log-rank test. Time to death was defined as the interval from waitlist removal to death or last follow-up. The Cox proportional hazards model was used to assess factors associated with overall survival post-waitlist removal, adjusting for relevant patient and clinical covariates. Effect modification by removal reason on the association between MASLD and overall survival was assessed by including an interaction term in the model. The proportional hazards assumption was evaluated using Schoenfeld residuals and the goodness-of-fit test described by Grambsch and Therneau [13].

All statistical analyses were performed using R version 4.5.1 (R Foundation for Statistical Computing, Vienna, Austria). A two-sided *p*-value < 0.05 was considered statistically significant.

## 3. Results

### 3.1. Demographics

A total of 6709 patients were evaluated for liver transplant in the CLN consortium, of which 1477 (22.0%) had MASLD and 5232 (78.0%) had non-MASLD etiologies. The demographics of each cohort are summarized in Table 1.

**Table 1:** Demographics.

Characteristic	Overall N = 6709 ^1^	Non-MASLD N = 5232 ^1^	MASLD N = 1477 ^1^	*p*-Value ^2^
Age at Referral	59 (50, 65)	57 (48, 64)	63 (56, 67)	<0.001
Sex				<0.001
female	2693 (40.1%)	1892 (36.2%)	801 (54.2%)	
male	4012 (59.8%)	3336 (63.7%)	676 (45.8%)	
Unknown	4	4	0	
Ethnicity				<0.001
Hispanic/Latino	2840 (43.8%)	2065 (40.9%)	775 (54.0%)	
Not Hispanic/Latino	3642 (56.1%)	2983 (59.0%)	659 (45.9%)	
Unknown	227	184	43	
Race				<0.001
African American	234 (3.7%)	219 (4.4%)	15 (1.1%)	
American Indian/Alaskan Native	65 (1.0%)	48 (1.0%)	17 (1.2%)	
Asian American/Pacific Islander	692 (10.9%)	589 (11.9%)	103 (7.3%)	
White	3706 (58.5%)	2847 (57.7%)	859 (61.3%)	
Other/Multiracial	1637 (25.8%)	1229 (24.9%)	408 (29.1%)	
Unknown	375	300	75	
Body Mass Index (BMI)	28 (24, 32)	27 (23, 31)	31 (27, 36)	<0.001
Unknown	51	41	10	
BMI Class				<0.001
Underweight	156 (2.3%)	136 (2.6%)	20 (1.4%)	
Normal	1999 (30.0%)	1801 (34.6%)	198 (13.4%)	
Overweight	2143 (32.2%)	1736 (33.4%)	407 (27.7%)	
Obese	1972 (29.6%)	1300 (25.0%)	672 (45.8%)	
Morbidly Obese	388 (5.8%)	218 (4.2%)	170 (11.6%)	
Unknown	51	41	10	
Decompensated Cirrhosis	4620 (68.8%)	3465 (66.2%)	1155 (78.2%)	<0.001
Hepatocellular Carcinoma	1530 (22.8%)	1217 (23.2%)	313 (21.2%)	0.094
Comorbidities				
Diabetes Mellitus	1748 (26.1%)	993 (19.0%)	755 (51.2%)	<0.001
Hypertension	2013 (30.0%)	1368 (26.1%)	645 (43.7%)	<0.001
Hyperlipidemia	848 (12.6%)	506 (9.7%)	342 (23.1%)	<0.001
Coronary Artery Disease	312 (4.7%)	213 (4.1%)	99 (6.7%)	<0.001

^1^ Median (Q1, Q3); n (%); ^2^ Wilcoxon rank sum test; Pearson’s Chi-squared test.

Patients in the MASLD cohort were significantly older than those in the non-MASLD cohort (median age 63 [56–67] years vs. 57 [48–64] years; *p* < 0.001). The MASLD group included a lower proportion of male patients (45.8% vs. 63.7%; *p* < 0.001). Racial and ethnic distribution differed significantly between groups (*p* < 0.001 for both). MASLD patients had a higher proportion of Hispanic/Latino patients (54.0% vs. 40.9%) than non-MASLD patients. The MASLD cohort had a lower proportion of African American (1.1% vs. 4.4%) and Asian American/Pacific Islander patients (7.3% vs. 12%), but a higher proportion of White patients (61.3% vs. 57.7%).

Body mass index (BMI) was significantly higher among MASLD patients (median 31 [27–36] vs. 27 [23–31]; *p* < 0.001). Correspondingly, obesity was more prevalent in the MASLD group, with higher rates of both obesity (BMI 30–39.9; 45.8% vs. 25.0%) and morbid obesity (BMI ≥ 40; 11.6% vs. 4.2%; *p* < 0.001). MASLD patients also had a higher prevalence of comorbid conditions, including diabetes mellitus (51.2% vs. 19.0%), hypertension (43.7% vs. 26.1%), hyperlipidemia (23.1% vs. 9.7%), and coronary artery disease (6.7% vs. 4.1%; *p* < 0.001 for all). Decompensated cirrhosis was more frequent in the MASLD cohort (78.2% vs. 66.2%; *p* < 0.001). Rates of hepatocellular carcinoma were similar between groups (21.2% vs. 23.2%; *p* = 0.094).

### 3.2. Predictors and Interaction Analyses of Waitlisting Among MASLD and Non-MASLD Candidates

Overall, 57.8% of MASLD and 48.3% of non-MASLD patients were waitlisted for transplant. A multivariable mixed-effects logistic regression model was performed to identify independent factors associated with waitlisting (Table 2). In this model, older age at referral was associated with slightly lower odds of waitlisting (OR 0.99 per year increase, 95% CI 0.99–1.00; *p* < 0.001). Sex and MELD at evaluation were not significantly associated with waitlisting. Compared with patients with normal BMI, those classified as morbidly obese had significantly lower odds of waitlisting (OR 0.65, 95% CI 0.52–0.82; *p* < 0.001), whereas other BMI categories were not significantly different.

The effect of relevant comorbidities (hypertension, coronary artery disease, diabetes, hyperlipidemia) was also evaluated. Patients with one comorbidity had slightly higher odds of being waitlisted compared to those without comorbidities (OR 1.15, 95% CI 1.01–1.30; *p* = 0.029), whereas patients with more than one comorbidity did not have significantly higher odds of being waitlisted. Importantly, MASLD etiology was independently associated with greater likelihood of waitlisting compared with non-MASLD etiologies (OR 1.52, 95% CI 1.33–1.74; *p* < 0.001).

To assess whether the association between liver disease etiology and waitlisting varied by BMI class, transplant center, or sex, interaction analyses were performed (Table 3). In the BMI interaction model, MASLD patients classified as overweight or obese had higher odds of being waitlisted compared with non-MASLD counterparts (overweight: OR 1.13, 95% CI 1.07–1.19; *p* < 0.001; obese: OR 1.12, 95% CI 1.07–1.17; *p* < 0.001). Normal-weight and underweight BMI classes showed no significant difference with waitlisting odds, and the overall interaction between BMI and etiology was also not significant (*p* for interaction = 0.096).

In the site interaction model, variation in odds of waitlisting across transplant centers was observed (*p* for interaction = 0.014). Sites 1, 2, 5 demonstrated significantly higher odds of waitlisting MASLD patients when compared to non-MASLD patients (site 1: OR 1.18, 95% CI 1.11–1.25; *p* < 0.001; site 2: OR 1.12, 95% CI 1.06–1.18; *p* < 0.001; site 5: OR 1.15, 95% CI 1.05–1.26; *p* = 0.002). In contrast, no significant interaction was observed between sex and etiology (*p* for interaction = 0.571). Odds of waitlisting were comparable between male and female candidates in both MASLD and non-MASLD groups.

### 3.3. Waitlist Outcomes

At five years after waitlist registration, approximately 15% of MASLD patients had died on the waitlist compared with 10% of non-MASLD patients, while rates of removal were similar between groups (both approximately 25%). The cumulative probability of liver transplantation by five years was 60–65% in both cohorts. Year-specific cumulative incidence rates for each outcome are shown in Supplemental Figure S1.

**Figure 2:**
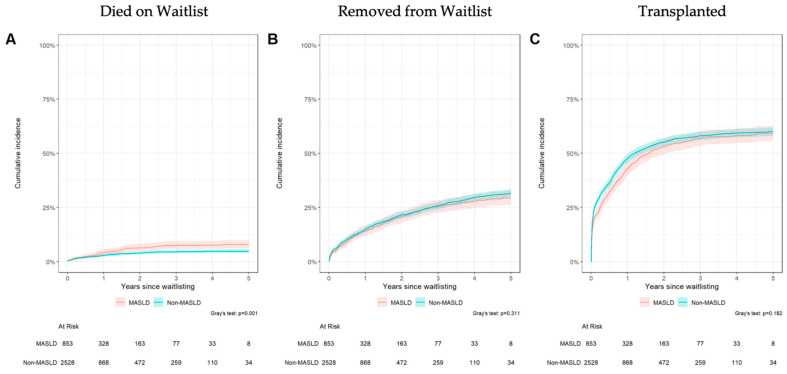
Cumulative Incidence analysis of waitlist mortality (**A**), removal from waitlist (**B**), and liver transplantation (**C**).

Competing-risk analysis demonstrated distinct cumulative incidence patterns for waitlist outcomes between MASLD and non-MASLD candidates (Figure 2). Patients with MASLD had a significantly higher cumulative incidence of waitlist death compared with non-MASLD candidates (*p* < 0.001; Figure 2A). Conversely, there was no significant difference in cumulative incidence of waitlist removal (*p* = 0.311; Figure 2B) or eventual liver transplantation (*p* = 0.192; Figure 2C).

Given the competing nature of transplant and death events, subsequent Fine–Gray competing-risk models were used to evaluate time-dependent outcomes of waitlist death, waitlist removal, and transplantation adjusting for covariates (Table 4). Older age at referral was independently associated with higher risk of death while on the waitlist (HR 1.02, 95% CI 1.00–1.03; *p* = 0.039), but not with waitlist removal or transplantation. Male candidates had a lower subdistribution hazard of waitlist death compared to females (HR 0.72, 95% CI 0.53–0.98; *p* = 0.036) but higher likelihood of transplantation (HR 1.19, 95% CI 1.06–1.32; *p* = 0.002).

Significant site variation was observed across transplant centers (*p* < 0.001). Compared with the reference site, Site 1 demonstrated higher hazards for death on the waitlist (HR 1.62, 95% CI 1.02–2.41; *p* = 0.017), while site 4 and 5 showed lower risk (site 4: HR 0.25, 95% CI 0.11–0.52; *p* = 0.002. Site 5: HR 0.45, 95% CI 0.23–0.88; *p* = 0.02). Furthermore, site 1 demonstrated significantly higher hazards for waitlist removal (HR 1.27, 95% CI 1.07–1.52; *p* = 0.007) and lower rates for transplantation (HR 0.67, 95% CI 0.57–0.58; *p* < 0.001). On the other hand, site 4, who has lower risk of waitlist death, also showed higher odds of proceeding with transplantation (HR 1.3, 95% CI 1.08–1.56; *p* = 0.005).

Among BMI categories, obesity was associated with an increased hazard of dying on waitlist compared with normal BMI (HR 1.52, 95% CI 1.00–2.30; *p* = 0.049), as well as lower odds for proceeding to transplantation (HR 0.86, 95%CI 0.75–0.99; *p* = 0.03). Other BMI groups did not show significant associations. Higher MELD score at listing was associated with mildly lower likelihood of being removed from waitlist (HR 0.97, 95% CI 0.97–0.98; *p* < 0.001) and greater likelihood of transplant (HR 1.06, 95% CI 1.05–1.07; *p* < 0.001).

Notably, in the adjusted Fine-Gray competing risk analysis, MASLD etiology was not independently associated with the subdistribution hazards of waitlist death, waitlist removal, or transplantation compared with non-MASLD etiologies.

### 3.4. MASLD Patients Have Lower Survival Rate After Waitlist Removal and Transplantation

**Figure 3:**
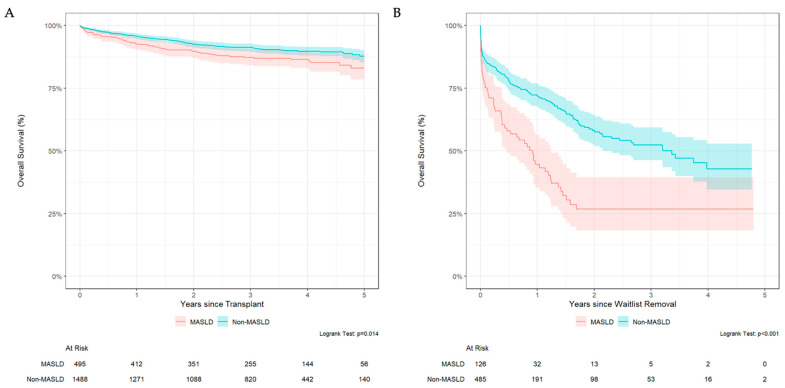
Kaplan–Meier Survival following liver transplantation (**A**) and for patients following waitlist removal (**B**).

Survival analyses were performed to evaluate post-waitlist removal and post-transplant survival among MASLD and non-MASLD patients. For patients who received a transplantation, MASLD recipients demonstrated lower 5-year post-transplant survival (83%, 95% CI 78–88%) compared to non-MASLD patients (88%, 95% CI 85–90%, log-rank *p* = 0.014; Figure 3A). In Cox analysis of post-transplant survival (Supplemental Table S2), MASLD remained a risk factor for inferior patient survival (HR 1.43; 95% CI 1.03–2.06; *p* = 0.033). Furthermore, in post-transplant survival analysis, when accounting for liver disease etiology, patients who were underweight had a significantly higher hazard ratio for mortality (HR 2.29; 95% CI 1.12–4.70; *p* = 0.02), while overweight patients had significantly lower hazard ratio for mortality (HR 0.66; 95% CI 0.46–0.96; *p* = 0.03).

Kaplan–Meier analysis for survival following waitlist removal revealed significantly reduced survival rates among MASLD candidates compared with non-MASLD candidates (log-rank *p* < 0.001; Figure 3B). In the adjusted multivariable Cox model, higher MELD at listing (HR 1.04, 95% CI 1.03–1.04, *p* < 0.001) and MASLD etiology (HR 1.64, 95% CI 1.14–2.35; *p* = 0.008) were significant predictors of mortality following waitlist removal (Supplemental Table S3). Age, sex, and BMI class were not significant predictors for mortality following waitlist removal. Regarding comorbidities, the presence of fewer than 4 comorbidities was not associated with mortality, while patients with four or more comorbidities demonstrated increased mortality risk following waitlist removal (HR 13.8, 95% CI 2.71–70.3; *p* = 0.002) although this finding may be confounded by small sample size given extremely wide confidence interval.

Additional Kaplan–Meier analyses stratified by reason of waitlist removal were shown in Supplemental Figure S1. When adjusting for covariates, the overall interaction between etiology and reason for waitlist removal was not significant (*p* for interaction = 0.809, Supplemental Table S4). However, subgroup analysis revealed higher mortality among MASLD compared with non-MASLD patients removed from the waitlist for clinical deterioration (HR 1.50, 95% CI 1.01–2.23; *p* = 0.044). No significant differences were observed for psychosocial (HR 1.63, 95% CI 0.47–5.59; *p* = 0.436), medical contraindication (HR 1.79, 95% CI 0.70–4.56; *p* = 0.221), condition improved (HR 4.88, 95% CI 0.29–83.35; *p* = 0.273), patient choice (HR 2.93, 95% CI 0.67–12.76; *p* = 0.152), or other reasons (HR 6.00, 95% CI 0.39–92.60; *p* = 0.199), noting wide confidence intervals consistent with small event counts in several strata.

## 4. Discussion

MASLD continues to grow as a leading indication for liver transplantation (LT), and the management of these patients throughout the transplantation process poses a challenge to the healthcare system [2]. Through this study of the California Liver Network, we identified that MASLD patients have higher rates of mortality on the waitlist, increased mortality following waitlist removal, and slightly impaired survival post-LT as compared to non-MASLD liver transplant candidates. The findings of this study identify areas of focus and potential intervention for MASLD candidates to optimize transplant outcomes in this growing cohort.

Consistent with existing literature, MASLD patients frequently present with a higher burden of medical comorbidities [6]. Specifically, MASLD patients are more likely to have history of diabetes, hypertension, hyperlipidemia and coronary disease as compared to non-MASLD LT candidates. Furthermore, MASLD candidates are more likely to present with decompensated cirrhosis at the time of listing. In all, these additional layers of complexity may help explain why MASLD patients have lower survival rate when removed from waitlist and even after transplant compared to non-MASLD patients. Although MASLD patients demonstrated higher waitlist mortality in this study, Fine-Gray analysis did not identify MASLD etiology as an independent predictor of waitlist mortality. This suggests that the ways MASLD physiology, associated comorbidities, and metabolic syndrome contribute to impaired survival across the transplant pathway are multifactorial. Regardless, there was still significant survival benefit after transplantation compared to patients who were removed from waitlist. This emphasizes the critical importance of timely access to transplantation for the MASLD cohort, even given their higher baseline risks. MASLD patients are likely to benefit from management of metabolic syndrome through weight loss and optimization of comorbidities as they progress through the transplant pathway. Pharmacologic agents such as glucagon-like peptide 1 (GLP-1) receptor agonists represent an emerging therapeutic class that may improve metabolic control, slow the progression of liver disease, and decrease risk for decompensation in MASLD patients, although the effects in cirrhotic patients and transplant recipients have not been fully investigated [14,15,16].

The effect of BMI on liver transplant candidacy also must be acknowledged. Our study demonstrated that morbid obesity (BMI ≥ 40) was associated with decreased likelihood for waitlisting overall. This reflects the technical complexity associated with performing a liver transplant in the morbidly obese recipient. Nonetheless, overweight (BMI 25.0–29.9) and obese (BMI 30–39.9) MASLD patients were more likely to be waitlisted than non-MASLD patients with similar BMI. Further, obese patients were more likely to die on the waitlist and less likely to proceed to transplant as compared to patients with normal BMI. In patients who were transplanted, underweight LT recipients (BMI < 18.5) demonstrated inferior post-transplant survival while overweight LT recipients demonstrated superior post-transplant survival. Frailty is known to be a negative predictor of post-transplant outcomes, and these results highlight the challenge in assessing frailty within the MASLD population. It is important to note that elevated BMI in chronic liver failure may be attributable to anasarca and/or fluid overload, which can mask underlying patient frailty. In fact, MASLD patients have a six-fold increased risk of having sarcopenic obesity [17]. One recent study demonstrated that for MASLD patients, lower BMI was associated with worse long-term graft and patient survival, in contrast to non-MASLD patients, where higher BMI was associated with worse survival [18]. While this study did not identify BMI as an overall predictor of post-transplant outcomes, studies have shown that patients with higher BMI were >10% more likely to be turned down for an organ [19]. These results suggest an inherent reluctance to transplant higher BMI candidates without clear evidence that they have worse post-transplant outcomes [18]. For patients whose BMI reflects obesity with potential technical challenge, weight loss approaches including glucagon-like-peptide 1 (GLP-1) receptor agonists and simultaneous liver transplant with sleeve gastrectomy are options to be considered to facilitate the technical aspects of transplantation, particularly in patients undergoing outpatient evaluation [20,21].

Transplant center site was an independent factor affecting waitlisting for both MASLD and non-MASLD patients. While an interesting finding, the underlying drivers of variance in practice between large academic institutions are likely multifactorial, including center-specific waitlisting practices and thresholds, differences in transplant candidate acuity and frailty, and differing approaches to managing medically complex patients. In the context of MASLD, we hope that the results of this study and further research help contribute to an informed, uniform approach to MASLD patients and associated comorbidities to guide waitlisting practices and ensure wider access to transplant.

This study likewise redemonstrated some gender-based disparity in access to liver transplant and post-transplant outcomes. In this study, there was no difference in waitlisting probability between male and female patients—distinct from previous studies demonstrating disparity in waitlisting for female patients [22]. However, the results of this study continued to demonstrate decreased rates of waitlist mortality and higher likelihood of transplant for male candidates as compared to female candidates. While sarcopenia and body habitus may be limiting factors in access to transplant and outcomes, this gender disparity should remain a focus in future studies.

A key strength of this study lies in the granular nature of its clinical data, which allowed for a detailed assessment of waitlist removal reasons and patient-level factors—information often sparse in large national databases. Additionally, the study population reflects greater racial and ethnic diversity compared to national cohorts, reflecting California’s demographic composition. However, this study is limited in its general applicability on a national scale, as California transplant centers represent higher median MELD at transplant than other regions throughout the United States [23]. Furthermore, the study period (2018–2020) overlapped with the early phase of the COVID-19 pandemic, which may have impacted listing and transplant practices. As longer-term follow-up data become available, it will be important to evaluate the consistency of these trends. Finally, the data source utilized in this study is limited, as donor and liver allograft data are not included in the analysis, which are known to impact transplant outcomes.

In summary, as MASLD becomes a more common indication for liver transplantation, it is imperative that perioperative and waitlist management strategies evolve to address the unique challenges of this patient population. Our findings support earlier identification and referral of MASLD patients before decompensation, integration of metabolic optimization during evaluation, and development of standardized criteria for MASLD patients to improve equity and transplantation access. Proactive optimization of comorbid conditions, a nuanced assessment of body composition rather than sole reliance on BMI, and multidisciplinary care are critical steps to improve waitlist outcomes and reduce mortality among MASLD patients.

## Figures and Tables

**Table 2:** Multivariable Logistic Regression for Waitlisting.

Characteristic	OR	95% CI	*p*-Value
Age	0.99	0.99, 1.00	<0.001
Sex			
female	—	—	
male	1.02	0.92, 1.13	0.8
BMI			
Normal	—	—	
Underweight	0.88	0.63, 1.23	0.4
Overweight	1.03	0.91, 1.17	0.7
Obese	1.08	0.95, 1.24	0.2
Morbidly Obese	0.65	0.52, 0.82	<0.001
MELD at evaluation	1.00	1.00, 1.01	0.14
Comorbidities Amount			
0	—	—	
1	1.15	1.01, 1.30	0.029
2	1.05	0.90, 1.23	0.6
3	0.94	0.75, 1.18	0.6
4	0.86	0.50, 1.46	0.6
Liver Disease Etiology			
Non-MASLD	—	—	
MASLD	1.52	1.33, 1.74	<0.001

Abbreviations: CI = Confidence Interval, OR = Odds Ratio.

**Table 3:** Logistic Interaction with Waitlisting with BMI, Site Interaction, and Sex.

	Estimate	*p*-Value		Estimate	*p*-Value		Estimate	*p*-Value
BMI		0.096	Site		0.014	Sex		0.571
Normal	1.08 (1.00, 1.16)	0.044	0	1.01 (0.95, 1.07)	0.834	Non-MASLD		
Underweight	1.20 (0.97, 1.50)	0.098	1	1.18 (1.11, 1.25)	<0.001	Male (vs. Female)	1.01 (0.98, 1.04)	0.573
Overweight	1.13 (1.07, 1.19)	<0.001	2	1.12 (1.06, 1.18)	<0.001	MASLD		
Obese	1.12 (1.07, 1.17)	<0.001	3	1.05 (0.95, 1.15)	0.317	Male (vs. Female)	0.99 (0.94, 1.04)	0.694
Morbidly Obese	0.98 (0.89, 1.08)	0.693	4	1.08 (0.99, 1.17)	0.091			
			5	1.15 (1.05, 1.26)	0.002			

**Table 4:** Fine-Gray Competing Risk Model for Waitlist Removal, Death, and Transplant.

	Died on Waitlist			Waitlist Removal			Transplanted		
Characteristic	HR	95% CI	*p*-Value	HR	95% CI	*p*-Value	HR	95% CI	*p*-Value
Age	1.02	1.00, 1.03	0.039	1.00	0.99, 1.00	0.7	1.00	1.00, 1.01	0.12
Sex									
female	—	—		—	—		—	—	
male	0.72	0.53, 0.98	0.036	0.95	0.84, 1.09	0.5	1.19	1.06, 1.32	0.002
Site									
0	—	—		—	—		—	—	
1	1.62	1.09, 2.41	0.017	1.27	1.07, 1.52	0.007	0.67	0.57, 0.78	<0.001
2	0.79	0.50, 1.25	0.3	1.00	0.83, 1.21	>0.9	1.09	0.94, 1.26	0.3
3	0.80	0.45, 1.44	0.5	1.00	0.79, 1.28	>0.9	1.08	0.91, 1.30	0.4
4	0.25	0.11, 0.59	0.002	0.94	0.74, 1.20	0.6	1.30	1.08, 1.56	0.005
5	0.45	0.23, 0.88	0.020	1.08	0.86, 1.37	0.5	1.04	0.87, 1.24	0.7
BMI									
Normal	—	—		—	—		—	—	
Underweight	1.03	0.31, 3.40	>0.9	1.10	0.72, 1.68	0.7	1.00	0.68, 1.46	>0.9
Overweight	1.49	0.99, 2.26	0.058	0.93	0.79, 1.09	0.4	0.93	0.82, 1.05	0.3
Obese	1.52	1.00, 2.30	0.049	1.01	0.86, 1.19	>0.9	0.86	0.75, 0.99	0.030
Morbidly Obese	1.58	0.79, 3.14	0.2	0.86	0.62, 1.19	0.4	1.08	0.86, 1.37	0.5
MELD at Listing	0.99	0.98, 1.01	0.4	0.97	0.97, 0.98	<0.001	1.06	1.05, 1.07	<0.001
Comorbidities Amount									
0	—	—		—	—		—	—	
1	0.91	0.61, 1.34	0.6	0.95	0.81, 1.10	0.5	1.09	0.97, 1.23	0.2
2	1.54	1.04, 2.28	0.031	0.99	0.82, 1.20	>0.9	0.94	0.81, 1.10	0.4
3	1.19	0.67, 2.13	0.6	0.92	0.69, 1.21	0.5	1.00	0.81, 1.22	>0.9
4	0.74	0.11, 5.17	0.8	0.38	0.14, 1.02	0.055	1.37	0.95, 1.97	0.10
Liver Disease Etiology									
Non-MASLD	—	—		—	—		—	—	
MASLD	1.20	0.87, 1.67	0.3	0.91	0.78, 1.07	0.3	1.00	0.89, 1.14	>0.9

Abbreviations: CI = Confidence Interval, HR = Hazard Ratio.

## Data Availability

The datasets presented in this article are not readily available because the data are part of ongoing studies and due to privacy issues. Requests to access the datasets should be directed to the corresponding author.

## Supplementary Materials

The following supporting information can be downloaded at: https://www.mdpi.com/article/10.3390/jcm14217841/s1, Table S1. Cumulative Incidence Table for Waitlist Death, Waitlist Removal and Transplantation. Table S2. Cox Model for Mortality Following Transplant. Table S3. Cox Model for Mortality Following Waitlist Removal. Table S4. Cox Model for Mortality Following Waitlist Removal with Interaction by Removal Reason. Figure S1. Kaplan–Meier Curves for Survival Following Waitlist Removal by Reason for Removal.

## Abbreviations

The following abbreviations are used in this manuscript:

MASLD	Metabolic dysfunction-associated steatotic liver disease
LT	Liver Transplantation
MELD	Model for End-Stage Liver Disease
BMI	Body Mass Index
CLN	California Liver Network
GLP-1	Glucagon-Like Peptide 1
