# Supplementary material for: Outcomes from Referral to Transplant for Patients with MASLD: A California Liver Network Study
Source: J Clin Med. 2025 Nov 4;14(21):7841. doi: 10.3390/jcm14217841 (PMC12608268; doi:10.3390/jcm14217841)

Supplemental Material

Table S1. Cumulative Incidence Table for Waitlist Death, Waitlist Removal and Transplantation

Time (years)	Non-MASLD	MASLD
Died on waitlist		
0	0.1% (0.0%, 0.3%)	0.0% (NA, NA)
1	2.9% (2.3%, 3.6%)	4.1% (2.9%, 5.5%)
2	4.0% (3.3%, 4.8%)	6.3% (4.8%, 8.1%)
3	4.5% (3.7%, 5.4%)	7.3% (5.7%, 9.2%)
4	4.7% (3.9%, 5.6%)	7.5% (5.8%, 9.4%)
5	4.8% (4.0%, 5.6%)	7.7% (6.0%, 9.7%)
Waitlist Removal		
0	0.5% (0.3%, 0.8%)	0.0% (NA, NA)
1	15.1% (13.8%, 16.6%)	14.0% (11.8%, 16.4%)
2	21.7% (20.2%, 23.4%)	20.4% (17.8%, 23.1%)
3	25.9% (24.2%, 27.7%)	25.0% (22.2%, 28.0%)
4	29.6% (27.8%, 31.5%)	27.4% (24.4%, 30.5%)
5	31.7% (29.8%, 33.6%)	28.9% (25.8%, 32.2%)
Transplanted		
0	0.9% (0.6%, 1.3%)	1.3% (0.7%, 2.2%)
1	47.8% (45.8%, 49.7%)	43.7% (40.3%, 46.9%)
2	55.3% (53.4%, 57.2%)	53.5% (50.1%, 56.8%)
3	58.2% (56.2%, 60.0%)	57.4% (54.0%, 60.7%)
4	59.4% (57.4%, 61.2%)	58.7% (55.2%, 61.9%)
5	60.0% (58.1%, 61.9%)	59.7% (56.2%, 63.0%)

Table S2. Cox Model for Mortality Following Transplant

Characteristic	HR	95% CI	p-value
Age	1.03	1.01, 1.04	**<0.001**
sex			
female	—	—	
male	1.3	0.96, 1.77	0.095
Site			
0	—	—	
1	1.53	0.96, 2.45	0.072
2	1.24	0.78, 1.98	0.400
3	1.5	0.86, 2.60	0.150
4	1.64	1.03, 2.60	**0.038**
5	1.16	0.62, 2.17	0.600
BMI			
Normal	—	—	
Underweight	2.29	1.12, 4.70	**0.024**
Overweight	0.66	0.46, 0.96	**0.032**
Obese	0.72	0.49, 1.05	0.088
Morbidly Obese	0.77	0.37, 1.58	0.500
MELD at Listing	1.00	0.99, 1.02	0.800
Comorbidities Amount			
0	—	—	
1	1.07	0.75, 1.51	0.700
2	1.03	0.65, 1.61	>0.900
3	1.16	0.63, 2.11	0.600
4	1.56	0.47, 5.16	0.500
Liver Disease Etiology			
Non-MASLD	—	—	
MASLD	1.46	1.03, 2.06	**0.033**

Abbreviations: CI = Confidence Interval, HR = Hazard Ratio

Table S3. Cox Model for Mortality Following Waitlist Removal

Characteristic	HR	95% CI	p-value
Age	1.00	0.99, 1.02	0.500
sex			
female	—	—	
male	1.04	0.76, 1.43	0.800
Site			
0	—	—	
1	2.44	1.45, 4.10	**<0.001**
2	2.36	1.48, 3.76	**<0.001**
3	3.07	1.75, 5.39	**<0.001**
4	2.79	1.37, 5.66	**0.005**
5	2.33	1.39, 3.90	**0.002**
BMI			
Normal	—	—	
Underweight	0.71	0.28, 1.80	0.500
Overweight	0.80	0.55, 1.16	0.200
Obese	1.11	0.76, 1.61	0.600
Morbidly Obese	1.80	0.74, 4.38	0.200
MELD at Listing	1.04	1.03, 1.06	**<0.001**
Comorbidities Amount			
0	—	—	
1	1.14	0.79, 1.64	0.500
2	1.38	0.93, 2.05	0.110
3	1.64	0.81, 3.33	0.200
4	13.8	2.71, 70.3	**0.002**
Waitlist Removal Reason			
Psychosocial	—	—	
Medical Contraindication	1.78	0.97, 3.27	0.061
Condition Deteriorated	3.28	2.04, 5.28	**<0.001**
Condition Improved	0.04	0.01, 0.18	**<0.001**
Patient Choice	0.60	0.25, 1.41	0.200
Other	0.18	0.04, 0.77	**0.022**
Liver Disease Etiology			
Non-MASLD	—	—	

**Characteristic**	**HR**	**95% CI**	**p-value**
MASLD	1.64	1.14, 2.35	**0.008**

Table S4. Cox Model for Mortality Following Waitlist Removal with Interaction by Removal Reason

Waitlist Removal Reason	Ratio	p-value
Psychosocial	1.629 (0.474, 5.592)	0.436
Medical Contraindication	1.789 (0.702, 4.56)	0.221
Condition Deteriorated	1.500 (1.011, 2.225)	0.044
Condition Improved	4.880 (0.286, 83.349)	0.273
Patient Choice	2.926 (0.671, 12.755)	0.152
Other	5.999 (0.389, 92.602)	0.199

P for interaction = 0.809

Figure S1. Kaplan-Meier Curves for Survival Following Waitlist Removal by Reason for Removal

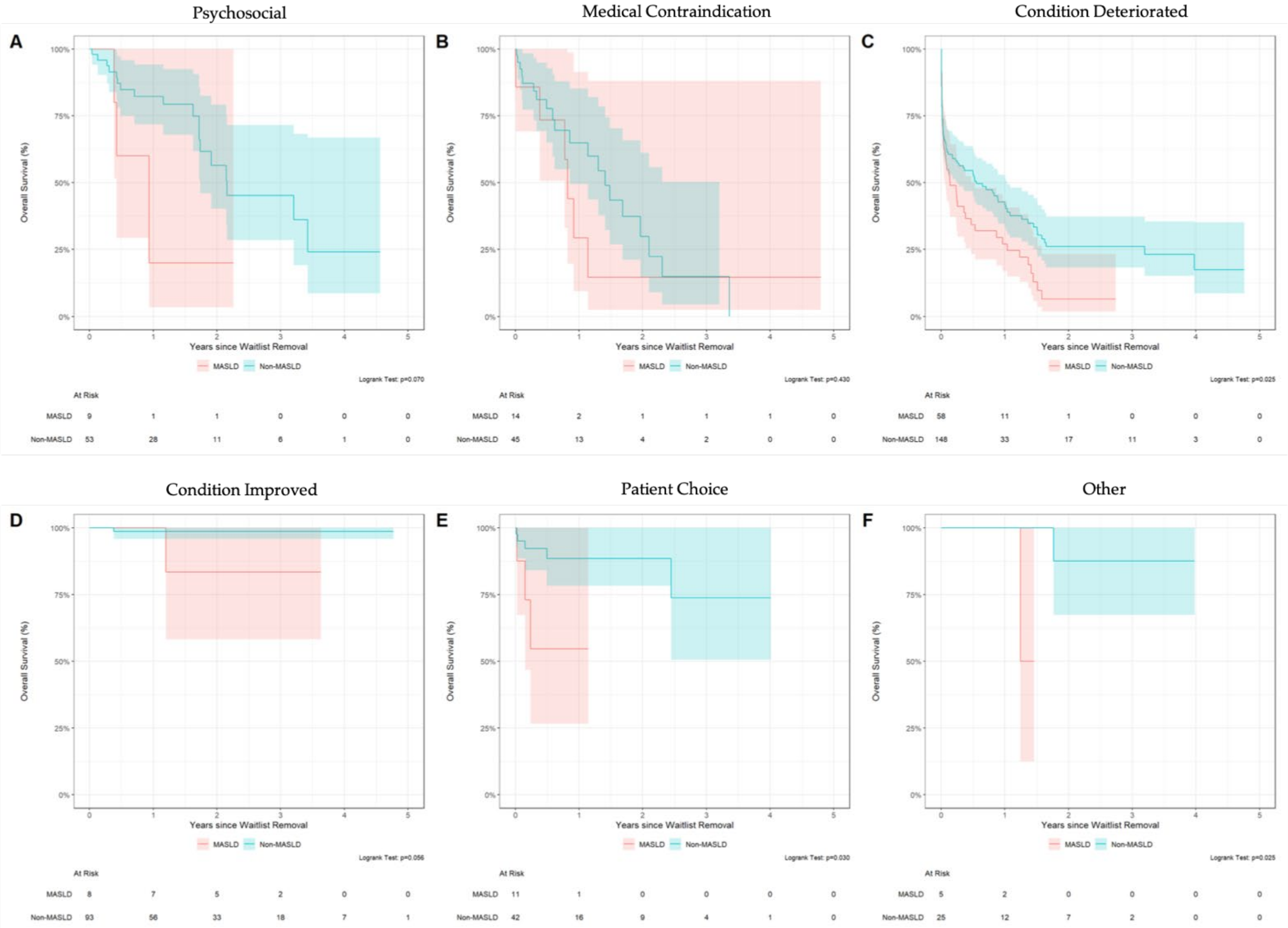

Supplement: Supplementary file 1 [file jcm-14-07841-s001.zip › jcm-3849990-supplementary.pdf]
